# Long Noncoding RNA NONHSAT233728.1 Promotes ROS Accumulation and Granulosa Cell Apoptosis by Regulating the MAPK/ERK1/2 Signaling Pathway

**DOI:** 10.1096/fj.202500964R

**Published:** 2025-05-28

**Authors:** Yao Chen, Heqi Dai, Fei Mao, Yangbai Li, Ruizhi Feng, Yun Qian

**Affiliations:** ^1^ Reproductive Medical Center of Second Affiliated Hospital of Nanjing Medical University Nanjing China; ^2^ State Key Laboratory of Reproductive Medicine Nanjing Medical University Nanjing China; ^3^ The Second Affiliated Hospital of Nanjing Medical University Nanjing China

**Keywords:** apoptosis, granulosa cell, lncRNA, polycystic ovary syndrome, reactive oxygen species

## Abstract

Polycystic ovary syndrome (PCOS) is one of the most prevalent endocrine disorders in women of reproductive age. However, the underlying molecular mechanism remains unclear. In this study, we employed RNA sequencing analysis to identify differentially expressed protein‐coding genes and long noncoding RNA (lncRNA) expression profiles in granulosa cells from women with and without PCOS. It was established that the level of NONHSAT233728.1 was diminished in women with PCOS. The present study demonstrated the role of NONHSAT233728.1 in granulosa cells from patients with PCOS and further investigated the potential mechanism of NONHSAT233728.1 in the KGN cell line. Additionally, the knockdown of NONHSAT233728.1 has been observed to promote cell apoptosis, inhibit cell proliferation, promote mitochondrial dysfunction, and inflammation. Western blot analyses confirmed that phospho‐extracellular regulated protein kinases (ERK)1/2 were decreased following lnc‐NONHSAT233728.1 knockdown. Consequently, we propose that ROS accumulation activates the endogenous mitochondrial apoptosis pathway, leading to granulosa cell apoptosis via the MEK/ERK1/2 pathway, which contributes to follicular atresia. We observed a negative correlation between NONHSAT233728.1 and both LH levels and the LH/FSH ratio. These findings indicate that lncRNA NONHSAT233728.1 is linked to the pathogenesis of PCOS and offer new insights into its underlying mechanisms.

## Graphical abstract

Through isolation and transcriptomic profiling of granulosa cells, we identified the functional significance of lncRNA NONHSAT233728.1 in polycystic ovary syndrome (PCOS) pathogenesis. Knockdown of lncRNA NONHSAT233728.1 in granulosa cells elevated ROS levels, reduced the Bcl2/Bax ratio, downregulated phospho‐ERK1/2, and increased IL‐8 secretion. These results demonstrate ROS‐mediated mitochondrial apoptosis via MEK/ERK1/2 suppression, alongside inhibited proliferation and enhanced inflammation, consistent with follicular atresia in PCOS. Negative correlations between NONHSAT233728.1 and LH/FSH ratios further support its pathogenic role.

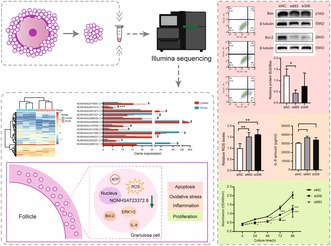

## 1 Introduction

Polycystic ovary syndrome (PCOS) is one of the most common endocrine disorders in women of reproductive age [1, 2], and is characterized by hyperandrogenemia, chronic oligomenorrhoea, and polycystic ovarian morphology [3]. PCOS is often associated with reproductive complications such as female infertility, ovulatory dysfunction, and pregnancy complications, as well as metabolic disorders such as insulin resistance and type 2 diabetes [4, 5]. Microarray data of granulosa cells from PCOS patients indicated that significantly altered genes were mainly associated with diabetes, inflammation, and oxidative stress [6]. Although the etiology and clinical symptoms of PCOS have been well studied, the potential molecular mechanisms remain unclear.

In patients with PCOS, the development of small ovarian follicles stops and remains in the sinus follicular stage with no dominant follicle formation, leading to persistent anovulation [7]. Granulosa cells provide nutrients and a suitable microenvironment during oocyte development, which are essential for follicular development and ovulation [8, 9]. A number of studies have shown that abnormal apoptosis and proliferation of ovarian granulosa cells in PCOS patients leads to early follicular maturation and subsequent atresia [10, 11, 12]. Thus, abnormal granulosa cell apoptosis promotes abnormalities in PCOS folliculogenesis; follicles in patients with PCOS are blocked at the small antral follicular stage, leading to anovulation and infertility [7]. Therefore, the abnormality of granulosa cells may be a critical factor in the pathogenesis of PCOS.

Redox homeostasis appears to be a critical factor in the normal functioning of mitochondria, cells, and organisms [13]. Oxidative stress, closely linked to reactive oxygen species (ROS) levels, is regulated by antioxidant mechanisms that maintain ROS at low concentrations, essential for normal cellular functions [14]. Excessive ROS can induce mitochondria‐mediated apoptosis [15]. Patients with PCOS often exhibit mitochondrial dysfunction and oxidative stress, characterized by increased ROS production [16, 17]. Granulosa cells from patients with PCOS exhibit mitochondrial dysfunction and oxidative stress‐induced apoptosis [18]. In addition, oxidative stress‐induced apoptosis significantly contributes to follicular atresia. Specifically, elevated ROS levels lead to premature ovarian failure and follicular atresia, and apoptosis is also heightened during follicular atresia [19]. Overall, oxidative stress induces granulosa cell apoptosis, thereby promoting follicular atresia, which may be a key factor in the pathogenesis of PCOS.

lncRNAs are transcripts longer than 200 nucleotides that lack protein‐coding capacity [20, 21]. They play diverse roles in biological processes such as cell development, differentiation, proliferation, and apoptosis [22, 23] by interacting with proteins, forming endogenous siRNA, regulating mRNA cleavage, chromatin remodeling, transcriptional regulation, and signal transduction [24, 25]. While abnormal expression of lncRNAs in granulosa cells of patients with PCOS has been reported in some studies, the specific roles and underlying mechanisms of these lncRNAs in PCOS remain poorly understood [26, 27, 28]. LncRNAs may participate in oocyte development, granulosa cell proliferation, and steroid production [29, 30, 31]. Furthermore, recent research has identified that specific lncRNAs are involved in physiological processes and pathological conditions, including human oocyte maturation, fertilization, and embryonic development, through their effects on granulosa cell function [30, 32].

In this study, we performed RNA sequencing analysis to identify differentially expressed protein‐coding genes and lncRNAs expression profiles in granulosa cells from women with and without PCOS. We found that lnc‐NONHSAT233728.1 was downregulated in women with PCOS. We demonstrated the role of NONHSAT233728.1 in granulosa cells and further investigated the potential mechanism of NONHSAT233728.1 in the KGN cell line. We then elucidated the possible molecular mechanisms focusing on oxidative stress‐induced apoptosis in granulosa cells. Our results demonstrated that LncRNA NONHSAT233728.1 promotes ROS accumulation and granulosa cell apoptosis by regulating the mitogen‐activated protein kinase (MAPK)/extracellular regulated protein kinases 1/2 (ERK1/2) pathway.

## 2 Materials and Methods

### 2.1 Subjects

Ovarian granulosa cells were collected from infertile patients who underwent in vitro fertilization/intracytoplasmic sperm injection‐embryo transfer (IVF/ICSI‐ET) at the Reproductive Center of the Second Affiliated Hospital of Nanjing Medical University from July 2020 to September 2021. The inclusion criteria for the PCOS group followed the Revised Rotterdam Diagnostic Criteria, which include ovulation disorder or anovulation; clinical and biochemical manifestations of hyperandrogenemia and polycystic ovary morphology. Patients meeting two or more criteria were diagnosed with PCOS and recruited into the study. Patients with organic and functional disorders of the hypothalamic–pituitary–ovarian axis, endocrine disorders, and drug‐induced hyperandrogenemia were excluded. All controls were women with regular menstrual cycles, normal ovulation, and sex hormone levels. A total of 46 patients were recruited into the study, with six samples for RNA sequencing and an additional 40 samples for RT‐qPCR validation. Clinical characteristics including age, BMI, endocrine, and biochemical parameters were recorded. Patients were informed about the study, signed an informed consent, and the study protocol was approved by the Ethics Committee of the Second Affiliated Hospital of Nanjing Medical University.

### 2.2 Isolation of Ovarian Granulosa Cells and Culture of KGN Cells

The gonadotropin‐releasing hormone (GnRH) agonist protocol (triptorelin, Lizhu, China) was used in both groups. After adequate follicular development as determined by both ovarian ultrasound and serum estradiol assay, patients received human chorionic gonadotropin (HCG, Lizhu, China), and ultrasound‐guided vaginal oocyte retrieval was performed 36 h later. Human ovarian granulosa cells were isolated from the patients' follicular fluid as previously described [33]. The human granulosa cell tumor‐derived cell line, KGN cells, was a gift from the research group of Prof. Ruizhi Feng. KGN cells were cultured in high glucose DMEM (C11995500BT, Gibco, Grand Island, NY) supplemented with 10% fetal bovine serum (SV30160.03, Hyclone, USA) and 1% penicillin–streptomycin (V900929, Sigma, USA) in a 37°C, 5% CO_2_ incubator. The cells were passed in a ratio of 1:2 to 1:3 every 2 days.

### 2.3 Total RNA Extraction (Human Ovarian Granulosa Cells) and RNA Quantification and Qualification

Total RNA was extracted from granulosa cells using the AllPrep DNA/RNA/miRNA Universal Kit (80224, Qiagen, Germany) according to the manufacturer's instructions. RNA purity and integrity were measured using the Nanodrop ND‐1000 (NanoDrop Technologies, Wilmington, USA) based on absorbance at 260 and 280 nm. RNA quality was assessed using the Agilent 2100 Bioanalyzer (Thermo Fisher Scientific, MA, USA).

### 2.4 Library Construction and Quality Control

Sequencing libraries were generated by Beijing BerryGenomics (Beijing, China) using the VAHTS mRNA‐seq v2 Library Prep Kit for Illumina according to the manufacturer's recommendations, and index codes were added to assign sequences to each sample. After library construction, the concentration of the library was measured using the Qubit fluorometer. The exact concentration of the cDNA library was checked again by qPCR. The size distribution of the library was determined by agarose gel electrophoresis.

### 2.5 RNA Sequencing Analysis

After library preparation and pooling of different samples, the samples were subjected to Illumina sequencing. PE150 (paired‐end 150nt) sequencing is commonly used for lncRNA‐seq. Raw data (raw reads) in FASTQ format were first processed by in‐house perl scripts to obtain clean data. Clean reads for each sample were first aligned to a reference genome using HISAT2 software. The read alignment results were subsequently transferred to the StringTie transcript assembly program. All transcripts were merged using Cuffmerge software. LncRNAs were then identified from the assembled transcripts. Quantification of transcripts and genes was performed using RSEM software to obtain reads per kilobase of transcript per million mapped reads (RPKM). EdgeR was used for differential expression analysis. The resulting *p*‐values were adjusted using the Benjamini and Hochberg approach to control for false discovery rate. Genes with |log_2_(fold change)| > 1 and *p*‐value < 0.05 were considered to be differentially expressed. Target gene prediction of lncRNAs was conducted via two approaches: cis‐acting target gene prediction and trans‐acting target gene prediction. Based on the cis‐acting regulatory element theory, the protein‐coding genes within 100 kb of the lncRNA were selected as potential cis‐acting targets. While for trans‐acting target prediction, the correlation coefficients between coding genes and lncRNAs were calculated, which required the sample size to be more than five.

### 2.6 Gene Ontology (GO) and Kyoto Encyclopedia of Genes and Genomes Functional Analyses (KEGG)

GO and KEGG enrichment analysis of differentially expressed gene sets was performed using the topGo R package and kobas2.0 software, respectively. Enrichment was considered significant when corrected *p*‐values were less than 0.05.

### 2.7 Transfection of Cells

KGN cells were seeded in 6‐well plates (2 × 10^5^ cells/well) or 24‐well plates (0.6 × 10^5^ cells/well) and incubated for 24 h. The cells were then transfected with 100 nM siRNAs (GenePharma, Shanghai, China) using HiPerFect Transfection Reagent (301705, Qiagen, Germany) following the manufacturer's protocol. The culture medium was replaced 24 h post‐transfection, and the cells were further incubated for 48 h before subsequent treatment. The sequences of siRNAs used are shown in Table S1.

### 2.8 Total RNA Extraction and Quantitative Real‐Time PCR

Total RNA from KGN cells was extracted using HiPure Total RNA Mini Kit (R4111‐02, Magen, China). All selected lncRNA primers were designed against the combined NCBI RefSeq (www.ncbi.nlm.nih.gov/refseq/) and NONCODE database (www.noncode.org) and synthesized by Shanghai Sangon Biotech Company (Sango, Shanghai, China). Then reverse transcribed into cDNA (HiScript III All‐in‐one RT SuperMix Perfect for qPCR, R333‐01, Vazyme, China) and target gene expression was detected by qRT PCR (Taq Pro Universal SYBR qPCR Master Mix, Q712‐02, Vazyme, China). The relative expression of RNA was calculated using the formula 2^−ΔΔCt^. GAPDH was used as a normalizer in RT PCR. The primer sequences of the genes tested are shown in Table S2.

### 2.9 Flow Cytometry Analysis

To detect apoptosis, KGN cells were harvested by adding trypsin without EDTA to 6‐well plates 48 h after transfection with siRNAs. Apoptosis staining was conducted according to the instructions of the Annexin V‐FITC/PI Apoptosis Detection Kit (A211‐02, Vazyme, China). The cells were washed twice with PBS and then resuspended with 100 μL of 1× binding buffer. Each group of samples was stained with 4 μL Annexin‐V‐FITC and 3 μL PI and incubated at room temperature in the dark for 10 min. Subsequently, 400 μL of 1× binding buffer was added to the cell suspension and flow cytometric analysis was performed after 1 h. The results were analyzed using FlowJo V10.

### 2.10 Cell Proliferation Assay

After transfection for 24 h, cells were harvested by trypsin. The CCK‐8 assay (A311‐01, Vazyme, China) was used to detect cell proliferation according to the manufacturer's protocol. The absorbance of the solution was measured at 450 nm.

### 2.11 Western Blot Assay

KGN cells were lysed in ice‐cold radioimmunoprecipitation assay (RIPA) buffer (P0013E, Beyotime Biotechnology, China) supplemented with 1% PMSF (ST506, Beyotime Biotechnology, China). Protein concentrations were measured by the BCA protein assay kit (P0012, Beyotime Biotechnology, China). Equal amounts of denatured protein samples were separated by SDS‐PAGE on a 10% gel and then transferred to polyvinylidene fluoride (PVDF) membranes. After blocking in 5% non‐fat milk, the membrane was incubated overnight at 4°C with primary antibodies against Bcl‐2 (1:2000, ab182858, Abcam, UK), Bax (1:5000, 60 267‐1‐Ig, Proteintech, USA) and β‐tubulin (1:4000, M30109, Abmart, China). After three washes in TBST buffer, the membranes were incubated with horseradish peroxidase‐conjugated secondary antibodies for 1 h with shaking. Protein bands were then visualized using High‐sig ECL Western Blotting Substrate (180‐501, Tanon, Shanghai, China).

### 2.12 Detection of Estrogen and Progesterone

Transfection 24 h later, the new medium was replaced and 10 nM testosterone (for estradiol analysis) (T102170, Aladdin, China) or 10 μM forskolin (for progesterone analysis) (F3917, Sigma, USA) was added to each well. After the cells were incubated for another 24 h, the culture medium was collected, centrifuged at 1100 rpm for 5 min, and the supernatant was retained and stored at −80°C. The levels of E2 and P4 of KGN cells in each group were detected by radioimmunoassay.

### 2.13 Inflammatory Factor Assay

Fresh medium was added 12 h after transfection. The supernatant was collected after 24 h of incubation, centrifuged at 1100 rpm, and diluted eight times. The levels of IL‐4, IL‐6, and IL‐8 were quantified using an iMatrix 100 luminometer.

### 2.14 Determination of ROS Levels

ROS levels were assessed using a Reactive Oxygen Species Assay Kit (S0033S, Beyotime Biotechnology, Shanghai, China). Transfected cells were incubated with 10 μmol/L 2′,7′‐dichlorodihydrofluorescein diacetate (DCFH‐DA) at 37°C for 20 min. The cells were washed three times with DCFH‐DA‐free PBS. Images were captured using a fluorescence microscope. Intracellular ROS levels in KGN cells were quantified with a Microplate Reader (BioTek Synergy2) at an excitation wavelength of 485 nm and an emission wavelength of 528 nm. The relative ROS levels were expressed as the fluorescence intensity of the siRNA group compared to the siNC group.

### 2.15 ATP Measurement

Cells were cultured in 24‐well plates and transfected. After 48 h, the culture medium was removed, and 150 μL of lysis buffer was added to each well to lyse the cells. The lysate was then centrifuged at 12 000×*g* for 5 min at 4°C, and the supernatant was collected for further analysis. Cellular ATP concentration was detected using an ATP assay kit (S0026, Beyotime Biotechnology, Shanghai, China) according to the manufacturer's instructions.

### 2.16 Subcellular Fractionation

KGN cells were harvested with trypsin and washed twice with PBS. Subcellular fractionation was performed using the Nuclear and Cytoplasmic Protein Extraction Kit (P0028, Beyotime Biotechnology, China), with RNase inhibitors (R0102, Beyotime Biotechnology, China) added during the procedure. RNA was then extracted from both cytoplasmic and nuclear fractions using RNA‐easy Isolation Reagent (R701‐01, Vazyme, China) and subjected to quantitative real‐time PCR. β‐Actin and nuclear U6 were used as markers for the cytoplasmic and nuclear fractions, respectively, in the quantitative real‐time PCR analysis.

### 2.17 Statistical Analysis

Statistical analyses were performed using GraphPad Prism 8.0 and SPSS 22. Data are expressed as the mean ± standard deviation (SD). Comparisons between groups were conducted using two‐tailed Student's *t*‐test for two‐group comparisons and one‐way ANOVA for multiple‐group comparisons. Spearman correlation analysis was employed to assess correlations. All experiments were conducted in triplicate, with *p < 0.05, **p < 0.01, ***p < 0.001indicating a significant difference.

## 3 Result

### 3.1 Evaluation of Clinical Characteristics

A total of 46 patients were included in this study, of which six cases (three control patients and three PCOS patients) were selected for transcriptome sequencing and the others (20 control patients and 20 PCOS patients) for RT‐qPCR. The main clinical characteristics are shown in Table 1. The serum levels of basal testosterone (T), basal luteinizing hormone (LH), LH/follicle‐stimulating hormone (FSH) and anti‐Müllerian hormone (AMH) were significantly elevated in the PCOS group, which was consistent with the typical features of PCOS.

**TABLE 1:** Clinical characteristics of PCOS and control groups.

	Cohort 1			Cohort 2		
	Control (*n* = 3)	PCOS (*n* = 3)	*p*	Control (*n* = 20)	PCOS (*n* = 20)	*p*
Age (years)	29 ± 3.61	29 ± 3.61	> 0.999	29.3 ± 3.06	27.6 ± 2.44	0.06
BMI (kg/m^2^)	23.3 ± 2.92	23.73 ± 4.03	0.8874	21.99 ± 2.94	24.68 ± 3.94	0.019*
Basal FSH (mIU/mL)	6.49 ± 0.15	7.69 ± 2.23	0.4066	7.25 ± 1.88	6.15 ± 1.49	0.049*
Basal LH (mIU/mL)	3.24 ± 0.96	11.39 ± 7.98	0.1538	3.94 ± 1.29	8.64 ± 4.47	< 0.0001****
LH/FSH	0.5 ± 0.16	1.37 ± 0.6	0.0731	0.55 ± 0.15	1.38 ± 0.57	< 0.0001****
Basal E2 (pg/mL)	39.33 ± 10.41	59 ± 45.25	0.4902	58.69 ± 41.49	80.82 ± 107.52	0.4
Basal T (ng/mL)	0.33 ± 0.08	0.64 ± 0.07	0.0067**	0.47 ± 0.13	0.74 ± 0.36	0.0028**
AMH (ng/mL)	3.6 ± 1.24	9.85 ± 1.98	0.0098**	3.8 ± 1.46	13.17 ± 4.41	< 0.0001****

*Note:* Indexes with significant differences (*p < 0.05, **p < 0.01, ****p < 0.0001).

### 3.2 Expression Profiles of Differentially Expressed Transcripts

**FIGURE 1:**
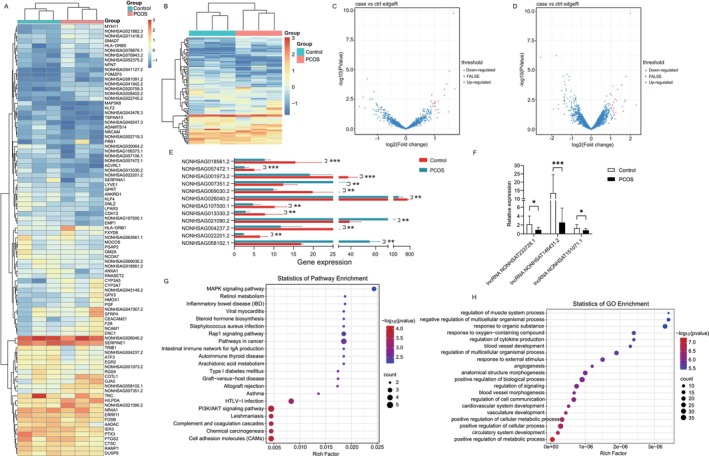
Differentially expressed lncRNAs and differentially expressed genes (DEGs) in granulosa cells from women with and without PCOS. (A, B) Heat map of the differentially expressed lncRNAs (fold change ≥ 2 and *p* value ≤ 0.05). (C) Volcano plot of differentially expressed lncRNAs. Among these lncRNAs, 16 lncRNAs were upregulated and 16 lncRNAs were downregulated. (D) Volcano plot of differentially expressed mRNAs. Among these mRNAs, 21 were upregulated and 37 were downregulated. (E) Twelve differentially expressed genes in RNA‐seq. The horizontal axis represents gene expression levels, while the vertical axis denotes the names of the lncRNAs. The data are presented with the control group indicated in red and the PCOS group in blue, *p < 0.05, **p < 0.05, ***p < 0.001. (F) The expression levels of three differentially expressed lncRNAs in a cohort of 20 PCOS patients and 20 controls using RT‐qPCR, *p < 0.05, **p < 0.05, ***p < 0.001. (G) KEGG pathway analysis on the mRNA expression data. (H) Gene Ontology (GO) functional analysis on biological processes. Data are expressed as the mean ± SD (*n* = 3).

To identify transcripts potentially involved in the etiology of PCOS, we performed high‐throughput sequencing to evaluate whole transcript expression profiles in granulosa cells from women with and without PCOS. A total of 32 lncRNAs and 58 protein‐coding transcripts exhibited differential expression between PCOS patients and controls (Figure 1A,B). Specifically, 16 lncRNAs were upregulated and 16 lncRNAs were downregulated (Figure 1C). Among the 58 differentially expressed mRNAs, 21 were upregulated and 37 were downregulated (Figure 1D). To focus on genes with significant expression differences, we conducted further analysis based on FPKM values (> 5). This analysis revealed 12 differentially expressed genes in granulosa cells, with nine genes downregulated and three genes upregulated in the PCOS group (Figure 1E). Among these 12 differentially expressed genes, we selected three that each correspond to a single transcript. The specific genes are NONHSAG107500.1, NONHSAG021090.2, and NONHSAG057472.1, with their transcript names being lncRNA NONHSAT233728.1, lncRNA NONHSAT146431.2, and lncRNA NONHSAT151071.1, respectively. To validate the sequencing results, we then verified their expression in a cohort of 20 PCOS patients and 20 controls using qRT‐PCR. We found that the expression levels of NONHSAT233728.1 and NONHSAT151071.1 were decreased in PCOS patients, consistent with the sequencing data (Figure 1F). However, the expression of NONHSAT146431.2 did not fully align with the sequencing results. The sequencing data have been uploaded to the relevant database (PRJNA1165653). To explore the potential functional role of lncRNAs in PCOS, we performed KEGG pathway analysis on the mRNA expression data. This analysis revealed that mRNAs were significantly enriched in 85 signaling pathways, with notable enrichment in pathways related to cell proliferation and apoptosis (Figure 1G). Among these, the PI3K/AKT signaling pathway was identified as highly relevant, and the MAPK signaling pathway was the most enriched. These findings suggest that lncRNAs may influence the development of PCOS through the two signaling pathways. We performed Gene Ontology (GO) functional analysis on differentially expressed lncRNAs (Figure S1A). The results indicated that these lncRNAs were predominantly involved in various biological processes, such as the positive regulation of cellular processes and the positive regulation of metabolic processes (Figure 1H). Cellular components were mainly enriched in extracellular structures and membrane components (Figure S1B). Additionally, molecular functions primarily included protein binding and receptor binding (Figure S1C).

### 3.3 Knockdown of lnc‐NONHSAT233728.1 Regulates Proliferation and Apoptosis of KGN Cells

**FIGURE 2:**
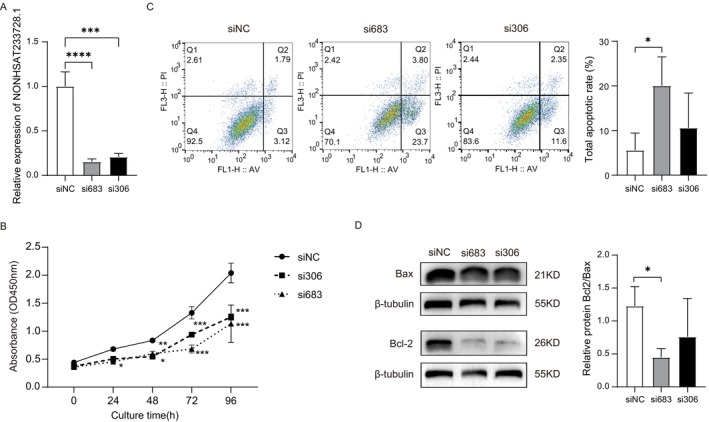
Knockdown of lnc‐NONHSAT233728.1 regulates proliferation and apoptosis of KGN cells. (A) Graph showing the expression level of lnc‐NONHSAT233728.1 in KGN cells treated with three different siRNA sequences via RT‐qPCR. The three distinct RNA sequences are siNC, si306, and si683, ****p < 0.0001. (B) Cell viability of KGN cells after transfection was measured by using Cell Counting Kit‐8, *p < 0.05, **p < 0.05, ***p < 0.001. (C) The apoptosis of transfected KGN cells was detected using Annexin V/PI staining, *p < 0.05. (D) The protein levels of Bax and Bcl‐2 in transfected KGN cells. Data are expressed as the mean ± SD (*n* = 3).

Since NONHSAT233728.1 was a previously unexplored lncRNA, its biological functions were unknown. Lnc‐NONHSAT233728.1 is located on chromosome chr14:61661250–61662046 and is highly expressed in the ovary and adrenal gland (http://www.noncode.org/show_gene.php?id=NONHSAG107500&version=1&utd=1#), suggesting a potential role in PCOS. To evaluate the effects of lnc‐NONHSAT233728.1 on cell biological function, KGN cells were transfected with two different siRNAs, si306 and si683, with a scrambled sequence used as the control. The knockdown efficiency of lnc‐NONHSAT233728.1 was confirmed by RT‐qPCR (Figure 2A). Cell proliferation was evaluated post‐transfection using CCK‐8 assays, which demonstrated that the knockdown of lnc‐NONHSAT233728.1 significantly inhibited cell proliferation in KGN cells (Figure 2B). Flow cytometry was used to assess apoptosis in KGN cells under each treatment condition. Early and late apoptotic cells were distributed in the Q3 and Q2 regions, respectively, while necrotic cells were in the Q1 region. Compared to the NC group, the apoptotic rate was significantly higher in the si683 group (*p* < 0.05, Figure 2C). Bcl‐2, an anti‐apoptotic protein, was downregulated in the siRNA treatment group (*p* < 0.05, Figure 2D). Additionally, the Bcl‐2/Bax ratio was significantly reduced in the siRNA treatment group, indicating a pro‐apoptotic effect. Western blot analyses further confirmed that knockdown of lnc‐NONHSAT233728.1 promotes apoptosis in KGN cells.

### 3.4 Knockdown of lnc‐NONHSAT233728.1 Regulates the Levels of Pro‐Inflammatory Cytokine and Sex Hormone In Vitro

**FIGURE 3:**
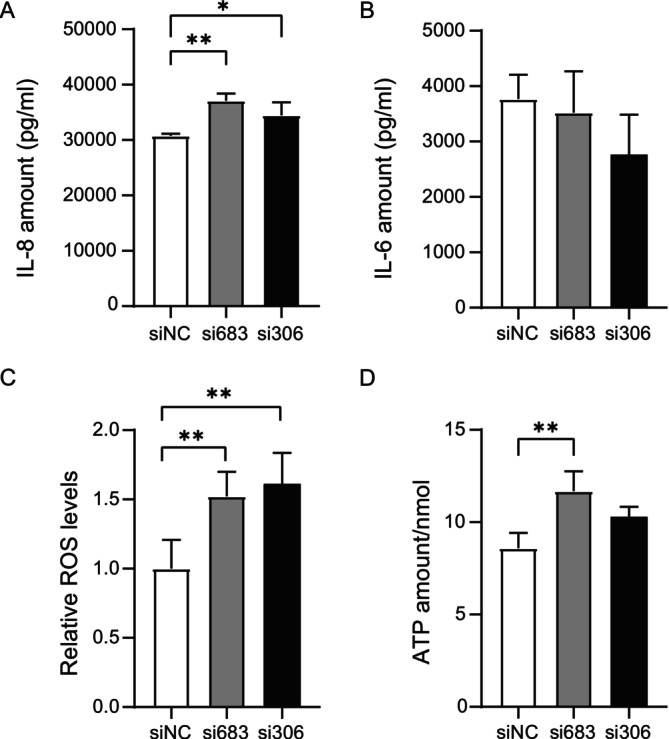
Knockdown of lnc‐NONHSAT233728.1 regulates pro‐inflammatory factor and impairs mitochondrial function in vitro. (A) Knockdown of lnc‐NONHSAT233728.1 upregulates the levels of IL‐8 in KGN cells, *p < 0.05, **p < 0.05. (B) Knockdown of lnc‐NONHSAT233728.1 upregulates the levels of IL‐6 in KGN cells, although no significant differences were observed between the samples. The expression of IL‐6 and IL‐8 was detected using an iMatrix 100 luminometer. (C) Knockdown of lnc‐NONHSAT233728.1regulates cell reactive oxygen species (ROS) generation in KGN cells, **p < 0.01. (D) The level of intracellular ATP was detected in transfected KGN cells, **p < 0.01. Data are expressed as the mean ± SD (*n* = 3).

Since the development of PCOS is accompanied by chronic inflammation, we measured the expression levels of IL‐4, IL‐6, and IL‐8. We found that the levels of the typical pro‐inflammatory cytokine IL‐8 were significantly increased in both the si683 and si306 treatment groups (Figure 3A), while the expression levels of IL‐6 and IL‐4 did not change significantly compared to the control group (Figure 3B, Figure S2). The results indicated that, compared to the siNC group, the levels of E2 were elevated in both the si683 and si306 groups, although these differences were not statistically significant (Figure S3A). The levels of P4 were significantly increased in the si683 group (*p* < 0.05, Figure S3B).

### 3.5 Knockdown of lnc‐NONHSAT233728.1 Impairs Mitochondrial Function In Vitro

As the Bcl‐2 protein family regulates apoptosis by controlling mitochondrial permeability, we further investigated its effects on mitochondrial function. We found that knockdown of lnc‐NONHSAT233728.1 led to a significant increase in ROS accumulation (Figure 3C). Additionally, adenosine triphosphate (ATP) concentrations in the si683 group were notably higher compared to controls (Figure 3D). Collectively, these results demonstrated that knockdown of lnc‐NONHSAT233728.1 impaired cellular mitochondrial function.

### 3.6 Identification and Subcellular Localization of lnc‐NONHSAT233728.1

**FIGURE 4:**
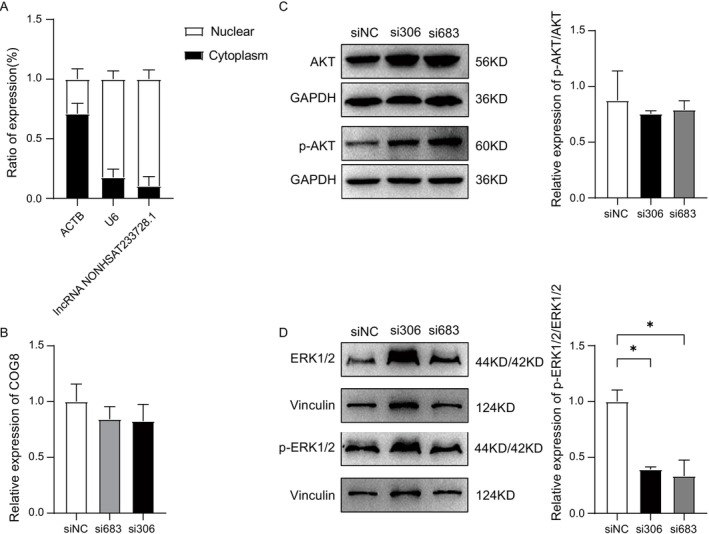
Subcellular localization and intrinsic mechanisms of lnc‐NONHSAT233728.1. (A) Nuclear and cytoplasmic fractions were analyzed using RT‐qPCR to determine the intracellular localization of NONHSAT233728.1. ACTB was used as a control for the cytoplasmic fraction, while U6 served as a control for the nuclear fraction. (B) The targeted mRNA expression of lncRNA NONHSAT233728.1. (C) The activity of PI3K/AKT signaling pathway was assessed by Western blot analysis following siRNA treatment. Protein levels of p‐AKT and AKT were evaluated using this method. (D) The activity of the MAPK/ERK1/2 signaling pathway was analyzed by Western blot after siRNA treatment. The protein levels of p‐ERK1/2 and ERK1/2 were detected by Western blot, *p < 0.05. Data are expressed as the mean ± SD (*n* = 3).

The subcellular localization of lncRNA plays a crucial role in the analysis of cellular functions and mechanisms [34]. To confirm the subcellular localization of NONHSAT233728.1 in KGN cells, RNA was extracted from nuclear and cytoplasmic fractions, and qRT‐PCR was performed. The results showed that NONHSAT233728.1 was mainly distributed in the nucleus, suggesting that it functions mainly in the nucleus (Figure 4A). LncRNAs typically regulate transcriptional programs in the nucleus through chromatin interaction and remodeling, and they can directly bind to target genes or recruit transcription factors to affect gene expression [35].

Additionally, through correlation analysis of lncRNA and mRNA expression in samples, we identified COG8 as a potential target of lncRNA NONHSAT233728.1. COG8 is a vesicular complex composed of eight proteins that is very important in regulating membrane transport and maintaining Golgi structure [36]. COG8 is essential for maintaining the integrity of the mammalian Golgi apparatus [37]. After knockdown of Inc‐NONHSAT233728.1, there was no significant difference in COG8 expression levels (Figure 4B). It is speculated that lnc‐NONHSAT233728.1 does not exert its effect by affecting COG8.

### 3.7 Knockdown of lnc‐NONHSAT233728.1 Promotes Apoptosis of Granulosa Cells via the MAPK/ERK1/2 Signaling Pathway

Various signaling pathways, including the phosphatidylinositol 3‐kinase (PI3K)/serine/threonine kinase (AKT) and MAPK/ERK1/2 pathways, are implicated in the pathogenesis of PCOS [38]. To explore their roles in cell apoptosis, we investigated the effects of lnc‐NONHSAT233728.1 knockdown. Knockdown of lnc‐NONHSAT233728.1 in KGN cells did not alter the protein levels of AKT or phosphorylated‐AKT (p‐AKT) (Figure 4C). However, while the total protein levels of ERK1/2 remained unchanged, the phosphorylation levels of ERK1/2 decreased following lnc‐NONHSAT233728.1 knockdown (Figure 4D). These results suggest that lnc‐NONHSAT233728.1 knockdown suppresses the activation of p‐ERK1/2, thereby influencing apoptosis.

### 3.8 Correlations Between NONHSAT233728.1 Expression and Clinical Parameters

**FIGURE 5:**
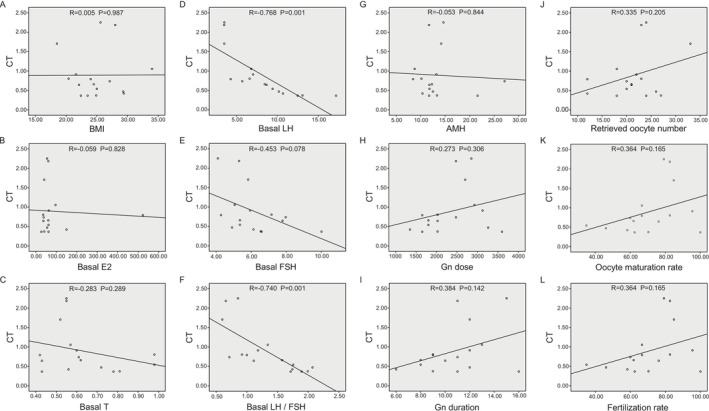
Correlations between NONHSAT233728.1 expression and clinical parameters. (A) Graph showing correlation between the relative expression of lnc‐NONHSAT233728.1 in granulosa cells of PCOS patients and BMI. (B) Graph showing correlation between the relative expression of lnc‐NONHSAT233728.1 in granulosa cells of PCOS patients and basal E2 levels. (C) Graph showing correlation between the relative expression of lnc‐NONHSAT233728.1 in granulosa cells of PCOS patients and Basal T levels. (D) Graph showing correlation between the relative expression of lnc‐NONHSAT233728.1 in granulosa cells of PCOS patients and Basal LH levels. (E) Graph showing correlation between the relative expression of lnc‐NONHSAT233728.1 in granulosa cells of PCOS patients and Basal FSH levels. (F) Graph showing correlation between the relative expression of lnc‐NONHSAT233728.1 in granulosa cells of PCOS patients and LH/FSH ratio. (G) Graph showing correlation between the relative expression of lnc‐NONHSAT233728.1 in granulosa cells of PCOS patients and AMH. (H) Graph showing correlation between the relative expression of lnc‐NONHSAT233728.1 in granulosa cells of PCOS patients with Gn dose. (I) Graph showing correlation between the relative expression of lnc‐NONHSAT233728.1 in granulosa cells of PCOS patients and Gn duration. (J) Graph showing correlation between the relative expression of lnc‐NONHSAT233728.1 in granulosa cells of PCOS patients and number of oocytes retrieved. (K) Graph showing correlation between the relative expression of lnc‐NONHSAT233728.1 in granulosa cells of PCOS patients and oocyte maturation rate. (L) Graph showing correlation between the relative expression of lnc‐NONHSAT233728.1 in granulosa cells of PCOS patients and fertilization rate. AMH, anti‐mullerian hormone; BMI, body mass index; E_2_, estradiol; FSH, follicle‐stimulating hormone; LH, luteinizing hormone; T, testosterone; oocyte maturation rate, number of oocytes at MII (metaphase II oocytes, 1st polar body) stage/total number of oocytes retrieved; Fertilization rate, number of fertilized embryos/total number of oocytes retrieved.

We examined the correlation between clinical characteristics of PCOS and the expression levels of lnc‐NONHSAT233728.1 in ovarian granulosa cells. Lnc‐NONHSAT233728.1 exhibited a significant negative correlation with LH concentration (Spearman's rho = −0.768, *p* = 0.001) and the LH/FSH ratio (Spearman's rho = −0.740, *p* = 0.001) in PCOS patients (Figure 5D,F). No significant correlations were observed between lnc‐NONHSAT233728.1 and other clinical parameters.

## 4 Discussion

In this study, we identified a total of 10 159 lncRNA transcripts from both PCOS and control groups, with 32 lncRNAs showing differential expression. Notably, we discovered a novel lncRNA, lnc‐NONHSAT233728.1, which appears to promote granulosa cell apoptosis through the MAPK/ERK signaling pathway. This finding highlights the potential role of lnc‐NONHSAT233728.1 in modulating apoptosis in PCOS.

The expression profile of NONHSAT233728.1 in human tissues reveals comparatively elevated expression levels in the ovary, suggesting that the ovary is its primary target organ. Our results demonstrated a significant decrease in the expression levels of NONHSAT233728.1 in patients with PCOS. Increasing evidence indicates that lncRNAs contribute to cell growth, development, and metabolic functions in human diseases [39, 40]. Specifically, lncRNAs are mainly involved in the pathogenesis of PCOS by affecting granulosa cell apoptosis, metabolic processes, and steroid hormone secretion [41, 42, 43]. Granulosa cells provide an essential microenvironment for follicle development and oocyte maturation, playing a critical role in ovarian follicle development. Numerous studies have shown that increased apoptosis of ovarian granulosa cells leads to follicular atresia [12, 44].

In this study, we measured the apoptosis of KGN cells in the siNC, si683, and si306 groups. Both the early and late apoptotic rates increased, and the ratio of Bcl‐2/Bax was significantly decreased in the siRNA treatment group. This indicated that downregulation of lnc‐NONHSAT233728.1 promoted apoptosis of granulosa cells. The anti‐apoptotic protein Bcl‐2 and the pro‐apoptotic protein Bax can regulate mitochondrial membrane permeability, thereby affecting mitochondrial redox metabolism [45]. Therefore, decreased Bcl‐2/Bax protein levels indicate mitochondrial‐dependent apoptosis.

Our results showed that knockdown of NONHSAT233728.1 led to a significant increase in ROS production and ATP levels. Previous reports have confirmed that excessive accumulation of ROS may cause mitochondrial dysfunction and granulosa cell apoptosis, which may affect oocyte energy supply and hinder oocyte maturation [46, 47]. Notably, oxidative stress can induce granulosa cell apoptosis, which is considered to be the main cause of follicular atresia [19]. Consequently, we speculated that knockdown of lnc‐NONHSAT233728.1 expression might damage mitochondrial function, activate the ROS‐dependent mitochondrial intrinsic apoptotic pathway, and contribute to follicular atresia.

In addition, the onset and development of PCOS are associated with chronic inflammation marked by elevated levels of pro‐inflammatory factors [48]. Studies have shown that the level of the pro‐inflammatory factor IL‐6 is increased in PCOS patients, with its regulatory mechanism linked to the overproduction of ovarian androgens [49]. Li et al. found that differentially expressed lncRNAs in PCOS patients were positively correlated with the levels of pro‐inflammatory factors [50]. In this study, we observed that knockdown of lnc‐NONHSAT233728.1 can positively regulate IL‐8 expression in KGN cells, suggesting that knockdown of lnc‐NONHSAT233728.1 may induce the inflammatory process.

LncRNA function is often associated with its subcellular localization [34]. To further verify the mechanism of lnc‐NONHSAT233728.1 in the pathogenesis of PCOS, we examined the subcellular localization of lnc‐NONHSAT233728.1 and found that it was predominantly located in the nucleus. This suggests that lnc‐NONHSAT233728.1 might exert its effects within the nucleus. Research indicates that lncRNAs frequently regulate transcription programs in the nucleus through chromatin interaction and remodeling and can bind directly to target genes or recruit transcription factors to affect gene expression [35]. We predicted COG8 as a potential target mRNA of lnc‐NONHSAT233728.1. However, knockdown of lnc‐NONHSAT233728.1 did not affect COG8 expression levels, suggesting that lnc‐NONHSAT233728.1 may not exert its effect by affecting COG8.

We conducted KEGG analysis on mRNA to further confirm the potential function of lncRNA in PCOS. The results indicate that mRNA is enriched in 85 signaling pathways. Notably, the pathways related to proliferation and apoptosis were significantly enriched. Among them, the PI3K/AKT pathway is crucial for the regulation of ovarian granulosa cell growth and follicle development [51]. Furthermore, there is evidence linking the PI3K/AKT pathway with the onset and progression of PCOS [52]. After knockdown of lnc‐NONHSAT233728.1, we detected the expression level of AKT and p‐AKT. Our results indicated no significant change in the ratio of p‐AKT to AKT, suggesting that lncRNA NONHSAT233728.1 does not affect granulosa cell growth via the PI3K/AKT pathway. The MAPK/ERK1/2 pathway is known to mediate cell proliferation and apoptosis [43]. Zhang et al. reported that increased ROS levels in hepatocellular carcinoma cells could promote apoptosis by inhibiting the MAPK/ERK1/2 pathway [53]. Han et al. demonstrated that the activated MAPK/ERK1/2 pathway inhibited apoptosis of ovarian granulosa cells [54]. Additionally, Liu et al. found that inhibition of the MAPK/ERK1/2 pathway promoted apoptosis of ovarian granulosa cells and increased ROS accumulation [55]. The MAPK/ERK1/2 pathway has been implicated in apoptosis induction through a ROS‐mediated mitochondrial pathway [53, 56].

**FIGURE 6:**
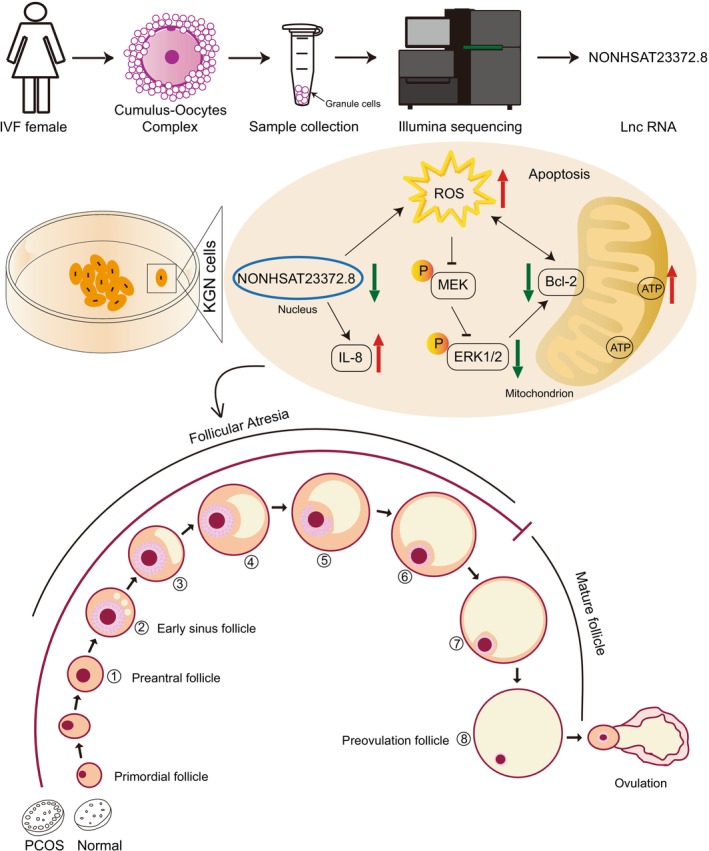
Schematic diagram for lnc‐NONHSAT233728.1function in women with PCOS. We employed RNA sequencing analysis to identify differentially expressed protein‐coding genes and long noncoding RNA (lncRNA) expression profiles in granulosa cells from women with and without PCOS. It was established that the level of NONHSAT233728.1 was diminished in women with PCOS. The present study demonstrated the role of NONHSAT233728.1 in granulosa cells from patients with PCOS and further investigated the potential mechanism of NONHSAT233728.1 in the KGN cell line. Knockdown of lnc‐NONHSAT233728.1 expression resulted in mitochondrial dysfunction and inflammation, characterized by the accumulation of reactive oxygen species (ROS). We hypothesize that this accumulation activates the endogenous mitochondrial apoptosis pathway, resulting in granulosa cell apoptosis through the MEK/ERK1/2 signaling pathway, which contributes to follicular atresia.

Therefore, we further investigated the MAPK/ERK1/2 pathway after the knockdown of lnc‐NONHSAT233728.1 in KGN cells. We found that the knockdown of lnc‐NONHSAT233728 led to a decrease in the ratio of p‐ERK1/2/ERK1/2. The results further confirmed our initial prediction based on the KEGG analysis. Specifically, the knockdown of lnc‐NONHSAT233728.1 induced apoptosis in KGN cells, accompanied by ROS accumulation and inhibition of the MAPK/ERK1/2 pathway. These findings suggest that the suppression of the MEK/ERK1/2 pathway may contribute to ROS‐induced apoptosis in KGN cells. We propose that ROS accumulation activates the endogenous mitochondrial apoptosis pathway, leading to granulosa cell apoptosis via the MEK/ERK1/2 pathway, which contributes to follicular atresia (Figure 6).

Knockdown of lnc‐NONHSAT233728.1 significantly promoted ROS‐associated apoptosis and inhibited the MEK/ERK1/2 pathway. However, the exact underlying mechanism remains unclear, and further in vitro and in vivo studies are required to elucidate these processes.

PCOS is a highly heterogeneous disorder, yet it may involve common pathological mechanisms underlying its diverse clinical manifestations. In our study, PCOS patients exhibited elevated levels of T, LH, LH/FSH, and AMH. Notably, we observed a negative correlation between NONHSAT233728.1 and both LH levels and the LH/FSH ratio. PCOS is characterized by elevated serum levels of LH, leading to hyperandrogenism and an altered LH to FSH ratio [57]. Elevated basal LH levels and an increased LH/FSH ratio are commonly observed in PCOS and are associated with infertility [58]. This suggests that NONHSAT233728.1 is associated with the clinical characteristics of PCOS and may play a role in its pathological processes. Therefore, it is valuable to expand the sample size and conduct a thorough investigation into the role of NONHSAT233728.1 in granulosa cells of women with PCOS, as well as its relationship with various clinical findings across different phenotypes.

In conclusion, our study revealed that lnc‐NONHSAT233728.1 is downregulated in ovarian granulosa cells from PCOS patients. The knockdown of NONHSAT233728.1 expression results in mitochondrial dysfunction and inflammation, characterized by reactive oxygen species (ROS) accumulation. We hypothesize that this accumulation triggers the endogenous mitochondrial apoptosis pathway, leading to granulosa cell apoptosis via the MEK/ERK1/2 signaling pathway, which subsequently contributes to follicular atresia. Additionally, we observed a negative correlation between NONHSAT233728.1 and both LH levels and the LH/FSH ratio. These findings indicate that lncRNA NONHSAT233728.1 is linked to the pathogenesis of PCOS and offer new insights into its underlying mechanisms.

## Author Contributions

Y.C. and Y.Q. conceived the study and supervised the analyses, Y.C. wrote, and R.F. revised and edited the manuscript. H.D. and F.M. conducted the experiments, Y.L. performed clinical sample collection, and R.F. provided experimental assistance. All authors contributed to study design, reviewing, and writing the manuscript. All authors critically reviewed and approved the final version of the manuscript.

## Conflicts of Interest

The authors declare no conflicts of interest.

## Supporting information


Figures S1–S3.

Tables S1–S2.

## Data Availability

All supporting data are included within the manuscript.
