# Supplementary material for: Long Noncoding RNA NONHSAT233728.1 Promotes ROS Accumulation and Granulosa Cell Apoptosis by Regulating the MAPK/ERK1/2 Signaling Pathway
Source: FASEB J. 2025 May 28;39(11):e70681. doi: 10.1096/fj.202500964R (PMC12117357; doi:10.1096/fj.202500964R)
Supplement: Supplementary file 1 — Figures S1–S3. [file FSB2-39-e70681-s002.docx]

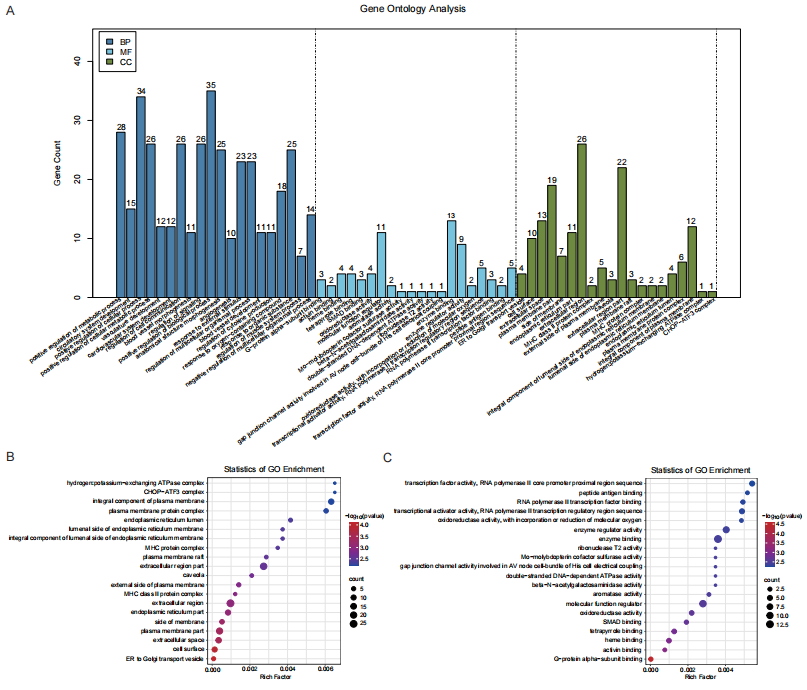


**Supplementary Fig. 1 Gene Ontology (GO) functional analysis. A** Bar diagram of GO functional enrichment analysis. **B** GO functional analysis on cellular components. **C** GO functional analysis on molecular functions.


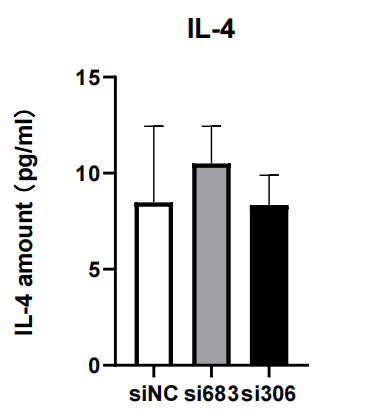


**Supplementary Fig. 2** The levels of IL-4 in KGN cell after siRNA treatment.


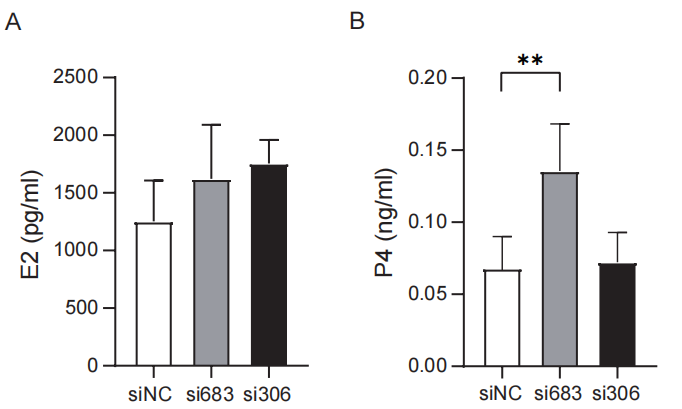


**Supplementary Fig. 3 Knockdown of lnc-NONHSAT233728.1 regulates the levels of sex hormone in vitro. A** No significant differences in E2 levels were observed in the siRNA-silenced groups (p > 0.05 ). **B** The levels of P4 were significantly increased in the si683 group, p < 0.05.
