# Supplementary material for: Long Noncoding RNA NONHSAT233728.1 Promotes ROS Accumulation and Granulosa Cell Apoptosis by Regulating the MAPK/ERK1/2 Signaling Pathway
Source: FASEB J. 2025 May 28;39(11):e70681. doi: 10.1096/fj.202500964R (PMC12117357; doi:10.1096/fj.202500964R)
Supplement: Supplementary file 2 — Tables S1–S2. [file FSB2-39-e70681-s001.docx]

**Supplementary Table 1. siRNA sequences used for transfection**

| **siRNA** | **Sequences** |
| --- | --- |
| si683-ss | CCCUGACAUUGUAUUAUAUTT |
| si683-as | AUAUAAUACAAUGUCAGGGTT |
| si306-ss | GGCGUCCUUUCCAUAUCUATT |
| si306-as | UAGAUAUGGAAAGGACGCCTT |
| siNC-ss | UUCUCCGAACGUGUCACGUTT |
| siNC-as | UUCUCCGAACGUGUCACGUTT |

**Supplementary Table 2. Primer sequences used for real-time PCR.**

| **primer** | **Sequences** |
| --- | --- |
| NONHSAT233728.1-F | AGGAGCCCATGAACCTAAGGA |
| NONHSAT233728.1-R | TATGGAAAGGACGCCTCTTGG |
| NONHSAT146431.2-F | GGTTTTCTCGGGGTGGCTT |
| NONHSAT146431.2-R | CGAGAATGGGAGAAAACGCAAA |
| NONHSAT151071.1-F | CAACAACAGCCTGACCCCTA |
| NONHSAT151071.1-R | GTGGGAAATCTCGCCTAAGGT |
| ACTB-F | CTCCATCCTGGCCTCGCTGT |
| ACTB-R | GCTGTCACCTTCACCGTTCC |
| U6-F | CTCGCTTCGGCAGCACA |
| U6-R | AACGCTTCACGAATTTGCGT |
| GAPDH-F | GGAGCGAGATCCCTCCAAAAT |
| GAPDH-R | GGCTGTTGTCATACTTCTCATGG |
| COG8-F | CGACTTGGCCTTCGCTAACTA |
| COG8-R | TAGGGTCAGGCTATTCATCCG |
